# Factors associated with loneliness in immigrant and Canadian-born older adults in Ontario, Canada: a population-based study

**DOI:** 10.1186/s12877-023-04092-w

**Published:** 2023-06-21

**Authors:** Mindy Lu, Susan E. Bronskill, Rachel Strauss, Alexa Boblitz, Jun Guan, James H.B. Im, Paula A. Rochon, Andrea Gruneir, Rachel D. Savage

**Affiliations:** 1 Dalla Lana School of Public Health, University of Toronto, Toronto, ON Canada; 2 ICES, Toronto, ON Canada; 3 Institute of Health Policy, Management and Evaluation, University of Toronto, Toronto, ON Canada; 4 Women’s Age Lab, Women’s College Hospital, 76 Grenville St, Toronto, ON M5S 1B2 Canada; 5 Department of Medicine, University of Toronto, Toronto, ON Canada; 6 Department of Family Medicine, University of Alberta, Edmonton, AB Canada

**Keywords:** Loneliness, Immigration, Population health, Canadian community health survey

## Abstract

**Background:**

While loneliness is common in older adults, some immigrant groups are at higher risk. To inform tailored interventions, we identified factors associated with loneliness among immigrant and Canadian-born older adults living in Ontario, Canada.

**Methods:**

We conducted a cross-sectional analysis of 2008/09 data from the Canadian Community Health Survey (Healthy Aging Cycle) and linked health administrative data for respondents 65 years and older residing in Ontario, Canada. Loneliness was measured using the Three-Item Loneliness Scale, with individuals categorized as ‘lonely’ if they had an overall score of 4 or greater. For immigrant and Canadian-born older adults, we developed separate multivariable logistic regression models to assess individual, relationship and community-level factors associated with loneliness.

**Results:**

In a sample of 968 immigrant and 1703 Canadian-born older adults, we found a high prevalence of loneliness (30.8% and 34.0%, respectively). Shared correlates of loneliness included low positive social interaction and wanting to participate more in social, recreational or group activities. In older immigrants, unique correlates included: widowhood, poor health (i.e., physical, mental and social well-being), less time in Canada, and lower neighborhood-level ethnic diversity and income. Among Canadian-born older adults, unique correlates were: female sex, poor mental health, weak sense of community belonging and living alone. Older immigrant females, compared to older immigrant males, had greater prevalence (39.1% vs. 21.9%) of loneliness.

**Conclusions:**

Although both groups had shared correlates of loneliness, community-level factors were more strongly associated with loneliness in immigrants. These findings enhance our understanding of loneliness and can inform policy and practice tailored to immigrants.

**Supplementary Information:**

The online version contains supplementary material available at 10.1186/s12877-023-04092-w.

## Background

Older immigrants are a rapidly growing population worldwide, primarily due to increasing rates of international migration as well as aging of immigrants who migrated earlier at young ages [1, 2]. This is especially true among host countries with long histories of immigration, like Canada, where one-third of older adults are foreign-born [3]. It is important to understand the unique needs of immigrants to ensure that services can effectively promote their health and well-being.

An important aspect of the older immigrant experience is social connection. Poor social connection may be reflected in measures of loneliness. Loneliness has been defined as an unpleasant feeling attributable to one’s perceived lack of quality or quantity of social relations [4], and is associated with adverse mental and physical health outcomes, including premature mortality [5–8]. Prior research indicates that older immigrants in North America and Europe experience disproportionately high prevalence of loneliness compared to their native-born counterparts [9–11]. Up to 54% of older immigrants experience feelings of loneliness, with older immigrant women experiencing significantly higher rates of loneliness than men [11–14].

Older immigrants experience unique post-migration stressors that may place them at higher risk for loneliness, above and beyond precipitating factors in later age like widowhood and chronic illness [3, 15]. Older immigrants frequently face loss of social support during migration, as well as post-migration socioeconomic disadvantages, language and cultural barriers, and experiences of discrimination that limit social opportunities [3, 16, 17]. Recent evidence shows that to address loneliness in older immigrants effectively, tailored solutions that reflect their unique risk factors are needed [18, 19]. Despite this, there is a limited understanding of which factors matter most as older immigrants are rarely studied on their own. Prior studies have identified country of birth, frequency of contact with others and living arrangement as factors of interest, although findings are inconsistent across studies [9, 13, 20, 21]. Additionally, there is limited research on older immigrant women, who face increased risks of loneliness compared to men due to differences in gender-based roles and experiences [14].

Therefore, we aimed to explore correlates of loneliness at the individual-, relationship-, and community-level in older immigrant men and women compared to their Canadian-born counterparts in Ontario, Canada to aid in the development of tailored and more effective solutions to loneliness in older adult immigrants.

## Methods

### Data sources and study participants

We analysed data from the Canadian Community Health Survey - Healthy Aging (CCHS-HA) cycle, a nationally representative cross-sectional survey in community dwelling individuals 45 years and older, which was administered by telephone between December 2008 and November 2009. A total of 30,865 individuals responded Canada-wide, representing a response rate of 74% [22]. Individuals were excluded from the survey if they: lived in Indigenous communities or collective dwellings (e.g., group home), were institutionalized (e.g., nursing home resident), or were a full-time Canadian Forces member [22].

CCHS-HA respondents who consented to linkage with provincial health administrative data and were Ontario residents aged 65 years and older were included (n = 2,671, Appendix A). The CCHS-HA sample was linked to population-based databases at ICES to characterize socio-demographic factors, neighbourhood-level characteristics and prior healthcare use. Datasets were linked using unique encoded identifiers and analysed at ICES. ICES is an independent, non-profit research institute whose legal status under Ontario’s health information privacy law allows it to collect and analyse health care and demographic data, without consent, for health system evaluation and improvement. Appendix B outlines the data sources and definitions of all included variables.

#### Immigration status

Immigration status was used to stratify the study sample into two groups. Respondents were asked on the CCHS-HA whether they were born a Canadian citizen. They were classified as Canadian-born if they responded ‘Yes’ and as an immigrant if they responded ‘No.’ Individuals with ‘Not Stated’ responses were excluded.

#### Primary outcome: loneliness

Loneliness was measured in the CCHS-HA using the Three-Item Loneliness Scale. This scale is used globally, valid in older adults interviewed by telephone, and has good internal consistency (α = 0.72) [23]. The participants responded to three questions that ask whether they feel: (a) lack of companionship, (b) left out, and (c) isolated from others. Each question is scored from 1 (hardly ever) to 3 (often), with an overall score between 3 and 9. Based on this score, prior studies have categorized individuals as experiencing no loneliness (score = 0–3), moderate loneliness (score = 4 or 5), or severe loneliness (score = 6–9) [24]. For this study, participants were categorized as ‘lonely’ if they had an overall score of 4 or greater to capture those experiencing any loneliness [24, 25]. Responses of ‘Don’t Know’, ‘Refusal’ or ‘Not Stated’ were treated as missing and excluded.

#### Correlates

Data analysis followed an ecological framework to acknowledge and address the importance of individual-, relationship- and community-level correlates of loneliness [26].

At the individual level, factors included socio-demographic characteristics, including age, sex and self-reported marital status, ethnicity and education level. Health status was assessed by measurement of self-perceived health (where health is defined by CCHS-HA interviewers as the lack of disease and injury, as well as physical, mental and social well-being), self-perceived mental health and number of primary care visits in the year prior. We considered having a valid driver’s license as a proxy for out-of-home mobility, as prior studies have shown that having a driver’s license is significantly associated with mobility among older adults [27]. Immigration-specific factors included self-reported country of birth, age at immigration, length of time in Canada since immigration and language ability. All of these variables were taken from CCHS-HA responses.

Relationship-level factors measure kin and non-kin relationships that influence loneliness [9]. These factors included household size, living arrangement, type of dwelling, positive social interaction score [28] and frequency of community-related activity participation. Feelings of wanting to participate more in social, recreational or group activities and sense of belonging to the local community were also considered.

One’s community plays an important role in shaping their ability to engage in social activities, which influence connectedness and loneliness [9]. Community-level factors included neighbourhood income quintile as well as dependency and ethnic diversity dimensions of marginalization within neighbourhoods [29]. These factors are measured for a dissemination area (400–700 people) and are based on the 2006 Canadian census.

### Statistical analysis

Descriptive analyses of the full range of factors were completed and stratified by immigration status using frequencies and means. Chi-squared tests and independent samples t-test were used to assess statistically significant differences between immigrant and Canadian-born groups for categorical and continuous measures of loneliness, respectively. A p-value of < 0.05 was considered statistically significant.

Separate multivariable logistic regression models were used to estimate adjusted odds ratios (aOR) and 95% confidence intervals (CI) and determine associations between loneliness and the factors of interest in immigrants and Canadian-born adults. Univariable analysis guided variable selection, with variables *P* < 0.20 included in multivariable analysis. The following factors were included in the regression model: age (continuous years); marital status; education level; self-perceived health; self-perceived mental health; number of primary care visits in the past year; has a valid driver’s license; time in Canada (continuous years); positive social interaction; frequency of community-related activity participation; wanted to participate more in social, recreational or group activities; sense of belonging to the local community; household size; living arrangement; type of dwelling; neighbourhood dependency score; neighbourhood ethnic diversity; and neighbourhood income.

Statistics Canada’s survey and bootstrap weights were incorporated in descriptive and regression analyses to obtain population-based frequencies, means and regression estimates [22]. All data analyses were completed using SAS Enterprise Guide software version 7.1 (SAS Institute Inc).

### Ethics approval

The use of the data in this project is authorized under Sect. 45 of Ontario’s Personal Health Information Protection Act (PHIPA) and does not require review by a Research Ethics Board (REB). Women’s College Hospital REB performed an administrative review (#2019-0148-E).

## Results

The weighted study population (n = 968 immigrants and 1,703 Canadian-born older adults) represented 1,401,764 people aged 65 or older. Baseline characteristics stratified by immigration status are presented in Table 1. At the individual level, older adult immigrants were more likely to be married (68.3% vs. 61.4%) and have more visits to primary care physicians (6.1 vs. 5.2 visits) but less likely to have a valid driver’s license (66.6% vs. 82.0%) and be of White ethnicity (72.6% vs. 99.5%), compared to their Canadian-born counterparts. At the relationship level, older immigrants had larger household sizes (2.3 vs. 1.8 people in the household) and were less likely to live alone compared to Canadian-born individuals (22.9% vs. 31.6%). At the community level, older immigrants were less likely to live in areas of the highest neighbourhood income quintile (17.5% vs. 19.7%) and more likely to live in areas of the highest ethnic diversity quintile compared to Canadian-born individuals (36.6% vs. 8.3%).

**Table 1:** Weighted* distribution of baseline characteristics of Canadian Community Health Survey (CCHS) respondents by immigration status

Characteristic	Canadian-Born	Immigrant
**Total Population**	N = 866146	N = 535618
**Sample Size**	n = 1703	n = 968
**Individual-Level Factors**		
** Age, mean**	74.4	74.4
** Sex (Female), %**	55.0%	52.0%
** Marital Status, %**		
Married or Common-Law	61.4%	68.3%
Separated or Divorced	7.9%	5.7%
Single, Never Married	3.4%	2.9%
Widowed	27.3%	23.1%
** Education Level, %**		
Less Than Secondary School	33.9%	36.2%
Secondary School	17.7%	20.0%
Some Post-Secondary School	6.1%	3.4%
Complete Post-Secondary School	42.2%	40.5%
** Ethnicity, %**		
White	99.5%	72.6%
Visible minority	0.5%	27.4%
** Self-Perceived Health, %**		
Good/Very Good/Excellent	78.6%	75.9%
Fair/Poor	21.4%	24.1%
** Self-Perceived Mental Health, %**		
Good/Very Good/Excellent	95.2%	94.4%
Fair/Poor	4.8%	5.6%
** Number of Primary Care Visits, mean**	5.2	6.1
** Have Valid Driver’s License (Yes), %**	82.0%	66.6%
**Relationship-Level Factors**		
** Household Size, mean**	1.8	2.3
** Living Arrangement (Living Alone), %**	31.6%	22.9%
** Type of Dwelling, %**		
Single Detached	65.4%	64.5%
Apartment	23.3%	21.9%
Other Multiple Dwelling Unit	10.4%	12.5%
Mobile Home or Other †	0.9%	1.1%
** Positive Social Interaction - Index Score, mean ‡**	13.6	13.3
** Frequency of Community-Related Activity Participation, %**		
Daily	14.2%	9.9%
Weekly	62.1%	63.3%
Monthly	16.7%	18.0%
Yearly	4.8%	5.3%
None	2.2%	3.4%
** Wanted to Participate More in Social, Recreational, Group Activities (Yes), %**	26.4%	24.5%
** Sense of Belonging to Local Community (Strong), %**	70.8%	67.1%
**Community-Level Factors**		
** Dependency Factor Score Quintile, % ‡**		
Quintile 1 (lowest)	7.6%	11.9%
Quintile 2	13.7%	18.6%
Quintile 3	19.5%	19.0%
Quintile 4	23.3%	22.0%
Quintile 5 (highest)	35.8%	28.5%
** Ethnic Diversity Factor Score Quintile, % ‡**		
Quintile 1 (lowest)	31.6%	16.9%
Quintile 2	24.1%	13.8%
Quintile 3	21.6%	15.8%
Quintile 4	14.5%	16.9%
Quintile 5 (highest)	8.3%	36.6%
** Neighbourhood Income Quintile, %**		
Quintile 1 (lowest)	18.2%	16.9%
Quintile 2	19.7%	24.0%
Quintile 3	22.6%	23.1%
Quintile 4	19.8%	18.5%
Quintile 5 (highest)	19.7%	17.5%

Note: Data were derived from the Ontario component of Canadian Community Health Survey (Healthy Aging) linked to health administrative databases. Characteristics grouped into three levels following the ecological framework: individual-level factors (i.e., sociodemographic, health status, transportation); relationship-level factors (i.e., factors measuring kin and non-kin relationships); community-level factors (i.e., measures of one’s surrounding environment).

* Weighted using Canadian Community Health Survey sampling weights and bootstrap weights provided by Statistics Canada.

† ‘Mobile home’ and ‘other’ groups suppressed to avoid small cells.

‡ Positive social interaction index score = One of four categories of social support measured by the Medical Outcomes Study Social Support Survey and measures the availability of other persons to positively interact with. The scale ranges from 0 to 16, with higher scores indicating higher level of positive social interaction.

Dependency factor score = A dimension of the Ontario Marginalization Index measuring area-level concentrations of people who do not have income from employment (e.g., older adults, children).

Ethnic diversity score = A dimension of the Ontario Marginalization Index measuring area-level concentrations of recent immigrants and/or racialized persons.

Immigration-related characteristics are presented in Table 2. Older immigrants were primarily long-standing immigrants, having lived in Canada for an average of 43.7 years. Over half were born in the United States or Europe. Of note, 14.5% of immigrants were unable to speak English or French.

**Table 2:** Weighted* distribution of immigration-related characteristics of Canadian Community Health Survey (CCHS) older immigrant respondents

Characteristic		Older Adult Immigrants
**Total Population**		N = 535,618
**Sample Size**		n = 968
**Time in Canada (Years)**	Mean (SE)	43.7 (0.9)
	Median (SE)	44.7 (1.2)
**Age at Immigration (Years)**	Mean (SE)	31.1 (0.9)
	Median (SE)	27.1 (0.7)
**Birth Country, %**		
US/UK/France		24.0%
Other Europe		32.2%
East Asia		6.5%
South Asia		7.5%
Southeast Asia		2.9%
South America		1.4%
Caribbean		2.7%
Other		22.7%
**Language able to Conduct a Conversation, %**		
(English or French) and Other		54.2%
English or French only		31.3%
Neither English nor French		14.5%

SE = standard error; UK = United Kingdom; US = United States.

Note: Data were derived from the Ontario component of Canadian Community Health Survey (Healthy Aging) linked to health administrative databases.

* Weighted using Canadian Community Health Survey sampling weights and bootstrap weights provided by Statistics Canada.

The level and prevalence of loneliness did not differ between immigrant and Canadian-born older adults overall or when further stratified by sex (Table 3). Older females – both immigrants and Canadian-born – had a substantially higher prevalence of loneliness (immigrant: 39.1% vs. 21.9%; Canadian-born: 41.0% vs. 25.7%) and greater severity of loneliness (immigrant: 3.9 vs. 3.5; Canadian-born: 3.9 vs. 3.6) than males.

**Table 3:** Weighted* loneliness estimates among Canadian Community Health Survey (CCHS) respondents by immigration status and sex

	Overall			Male			Female		
Level	Canadian-Born	Immigrant	P-Value	Canadian-Born	Immigrant	P-Value	Canadian-Born	Immigrant	P-Value
**Total Population**	866,146	535,618		389,829	257,351		476,316	278,267	
**Sample Size**	1703	968		690	417		1013	551	
**Loneliness Score, mean (SE)**	3.8 (0.0)	3.7 (0.0)	0.4976	3.6 (0.0)	3.5 (0.1)	0.8151	3.9 (0.1)	3.9 (0.1)	0.6371
**Any Loneliness Status, %**									
Lonely	34.0%	30.8%	0.1858	25.7%	21.9%	0.2280	41.0%	39.1%	0.5981
Not Lonely	66.0%	69.2%		74.3%	78.1%		59.0%	60.9%	

Note: Data were derived from the Ontario component of Canadian Community Health Survey (Healthy Aging) linked to health administrative databases.

* Weighted using Canadian Community Health Survey sampling weights and bootstrap weights provided by Statistics Canada.

**Fig. 1:**
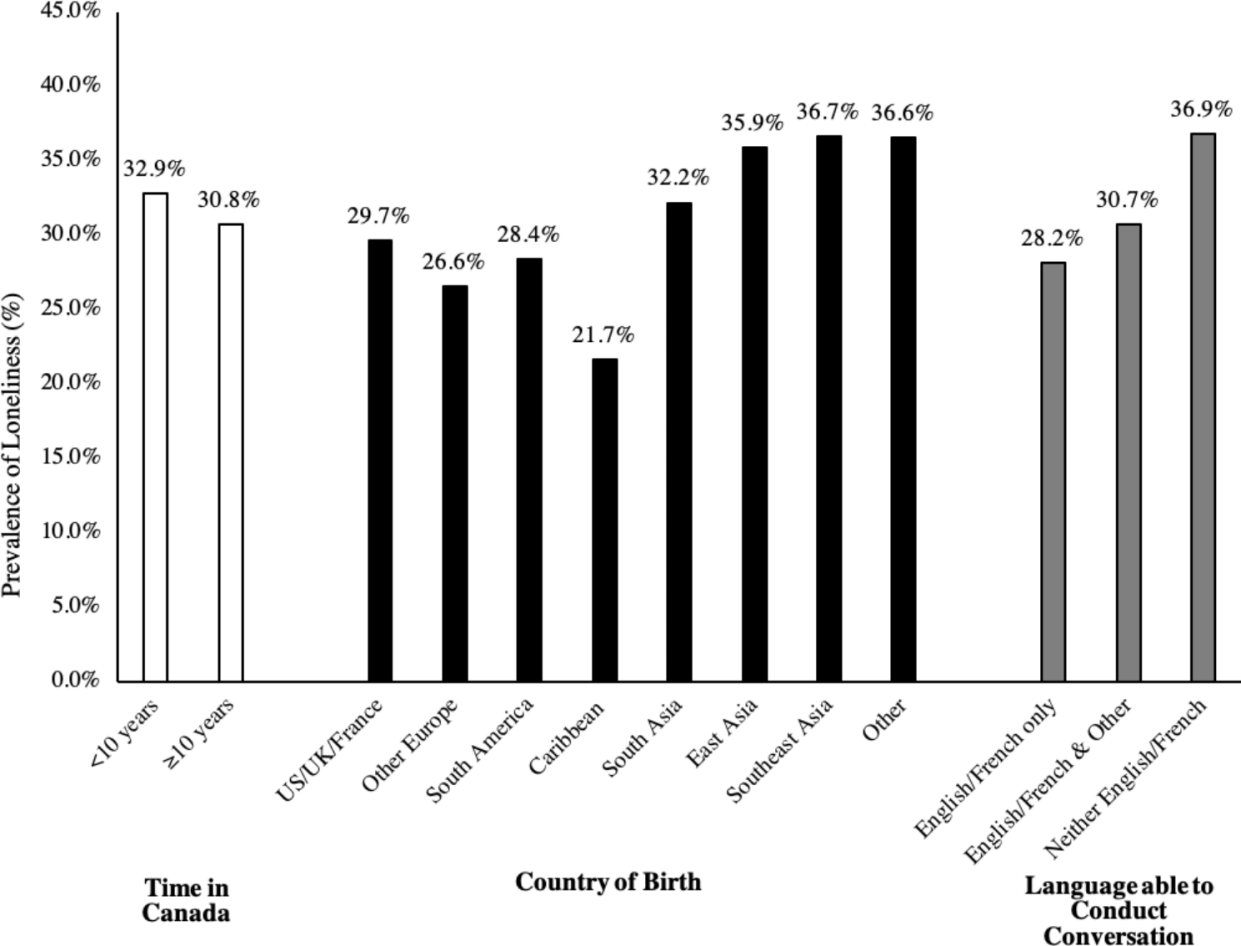
Distribution of loneliness prevalence by immigration-related characteristics. Immigrant respondents to the Canadian Community Health Survey (CCHS) between 2008 and 2009, aged 65 years and older residing in Ontario, Canada, weighted using CCHS sampling weights and bootstrap weights provided by Statistics Canada

As presented in Fig. 1, the prevalence of loneliness among immigrants varied by time in Canada, country of birth and language ability. Recent immigrants (within 10 years) had a higher prevalence of loneliness compared to long-term immigrants (32.9% vs. 30.8%). Immigrants born in Asia had a higher prevalence of loneliness (range: 32.2–36.7%) than those born in the United States, Europe, South America or the Caribbean (range: 21.7–29.7%). Immigrants who spoke neither of Canada’s official languages (English or French) had a higher prevalence of loneliness compared to those who spoke English or French but no additional languages (36.9% vs. 28.2%).

Table 4 presents the multivariable regression analyses. Among older adult immigrants, being widowed and having fair or poor self-perceived health were both associated with an approximate 2-fold increase in the odds of loneliness (marital status aOR 1.98, 95% CI 1.08–3.62; self-perceived health aOR 1.85, 95% CI 1.24–2.75). A longer duration in Canada was associated with lower odds of loneliness, with a 2% reduction in the odds of loneliness for each year spent in Canada (aOR 0.98, 95% CI 0.97–0.99). Relationship-level factors were also important in predicting loneliness among older immigrants. A one-point increase in positive social interaction score was associated with a 21% decrease in loneliness odds (aOR 0.79, 95% CI 0.75–0.84). Feelings of wanting to participate more in social, recreational or group activities were associated with almost 4-times the odds of loneliness compared to those without such feelings (aOR 3.60, 95% CI 2.34–5.54). At the community-level, living in neighbourhoods of increased ethnic diversity decreased the odds of loneliness (aOR 0.78, 95% CI 0.66–0.93), while living in an area within the lowest quintile of neighbourhood income increased the odds of loneliness (aOR 2.04, 95% CI 1.04–3.98).

**Table 4:** Fully adjusted odds ratios comparing odds of loneliness among immigrant and Canadian-born older adults

Variable	Immigrant (aOR, 95% CI)	Canadian-Born (aOR, 95% CI)
**Individual-Level Factors**		
** Age**	1.02 (0.99–1.04)	1.00 (0.99–1.02)
** Sex**		
Male	(ref)	(ref)
Female	1.42 (0.93–2.18)	1.50 (1.13–1.99)**
** Marital Status**		
Married or Common-Law	(ref)	(ref)
Separated or Divorced	2.14 (0.99–4.61)	1.73 (1-2.99)
Single, Never Married	0.78 (0.26–2.34)	1.13 (0.54–2.38)
Widowed	1.98 (1.08–3.62)*	1.55 (0.94–2.55)
** Education Level**		
Less Than Secondary School	0.80 (0.52–1.23)	1.07 (0.8–1.43)
Secondary School	0.71 (0.43–1.16)	1.11 (0.74–1.67)
Some Post-Secondary School	0.56 (0.20–1.55)	0.77 (0.43–1.38)
Completed Post-Secondary School	(ref)	(ref)
** Self-Perceived Health**		
Good/Very Good/Excellent	(ref)	(ref)
Fair/Poor	1.85 (1.24–2.75)**	1.22 (0.88–1.70)
** Self-Perceived Mental Health**		
Good/Very Good/Excellent	(ref)	(ref)
Fair/Poor	0.80 (0.37–1.73)	2.91 (1.57–5.40)***
** Number of Primary Care Visits in Past Year**	1.02 (0.99–1.06)	0.99 (0.96–1.01)
** Have Valid Driver’s License**		
No	(ref)	(ref)
Yes	0.89 (0.57–1.39)	1.06 (0.75–1.5)
** Time in Canada**	0.98 (0.97–0.99)**	-
**Relationship-Level Factors**		
** Positive Social Interaction - Index Score**	0.79 (0.75–0.84)***	0.82 (0.79–0.86)***
** Frequency of Community-Related Activity Participation**		
Daily	(ref)	(ref)
Weekly	0.78 (0.43–1.41)	0.79 (0.54–1.15)
Monthly	0.87 (0.43–1.75)	1.01 (0.62–1.66)
Yearly	0.97 (0.41–2.31)	0.79 (0.39–1.62)
None	0.63 (0.18–2.14)	1.08 (0.39–2.98)
** Wanted to Participate More in Social, Recreational, Group Activity**		
No	(ref)	(ref)
Yes	3.60 (2.34–5.54)***	3.65 (2.78–4.79)***
** Sense of Belonging to Local Community**		
Strong	(ref)	(ref)
Weak	1.01 (0.69–1.48)	1.41 (1.06–1.88)*
** Household Size**	0.93 (0.74–1.17)	1.26 (0.84–1.88)
** Living Arrangement**		
Living with Others	(ref)	(ref)
Living Alone	1.73 (0.87–3.42)	2.60 (1.44–4.70)**
** Type of Dwelling**		
Single Detached	(ref)	(ref)
Apartment	0.74 (0.45–1.20)	0.89 (0.63–1.27)
Mobile Home	0.87 (0.05–15.70)	1.27 (0.48–3.39)
Other Multiple Dwelling Unit	1.06 (0.62–1.81)	0.95 (0.61–1.48)
Other	0.62 (0.00-164.91)	0.28 (0.02–3.59)
**Community-Level Factors**		
** Dependency Factor Score**	0.92 (0.71–1.19)	1.06 (0.93–1.29)
** Ethnic Diversity Factor Score**	0.78 (0.66–0.93)**	0.88 (0.75–1.04)
** Neighbourhood Income Quintile**		
Quintile 1	2.04 (1.04–3.98)*	0.84 (0.51–1.39)
Quintile 2	1.46 (0.82–2.59)	1.06 (0.68–1.66)
Quintile 3	0.98 (0.50–1.94)	0.98 (0.63–1.54)
Quintile 4	1.67 (0.94–2.98)	0.73 (0.47–1.12)
Quintile 5	(ref)	(ref)

aOR = adjusted odds ratio; CI = confidence interval

Note: Data were derived from the Ontario component of Canadian Community Health Survey (Healthy Aging) linked to

health administrative databases, weighted using Canadian Community Health Survey sampling weights and bootstrap weights provided by Statistics Canada.

* *P* < 0.05; ** *P* < 0.01; ***P < 0.001

Among Canadian-born older adults, female sex (aOR 1.50, 95% CI 1.13–1.99), self-perceived mental health (aOR 2.91, 95% CI 1.57–5.40), positive social interaction (aOR 0.82, 95% CI 0.79–0.86), feelings of wanting to participate more (aOR 3.65, 95% CI 2.78–4.79), weak sense of belonging (aOR 1.41, 95% CI 1.06–1.88) and living alone (aOR 2.60, CI 1.44–4.70) were independent risk factors for loneliness. There were no significant correlates of loneliness for Canadian-born older adults at the community level.

## Discussion

This study identified common and unique correlates of loneliness among older immigrants and Canadian-born individuals living in Ontario, Canada. Unlike other studies, our analysis considered community-level factors, in addition to individual- and relationship-level factors, to acknowledge the complex nature of loneliness. We found that community-level correlates were more strongly associated with loneliness in older immigrants than in Canadian-born participants.

Over one third of older adults reported feelings of loneliness, with no significant differences in loneliness between older immigrants and older Canadian-born individuals. This is contrary to findings from previous Canadian studies, which report a higher mean level of loneliness in all immigrant older adults compared to their Canadian-born counterparts [9, 20]. Our findings may be explained in part by the immigrant participants included in the study sample, who were mostly long-standing immigrants from the United States or Europe. Previous studies have found that greater cultural and linguistic distance between the home and host country is associated with increased loneliness [30]. For example, upon stratifying immigrants by origin, De Jong et al. [9] found greater loneliness in older migrants to Canada than non-migrants, except for those who migrated from Britain or France. We similarly found that the prevalence of loneliness was higher among immigrants from countries of origin with greater cultural distance (i.e., higher among immigrants from Asia compared to the United States or Europe). We also found greater loneliness among immigrants with greater linguistic distance (i.e., immigrants who spoke neither of Canada’s official languages). These findings speak to the importance of exploring diversity within immigrant populations in loneliness research and repeating studies over time as immigration patterns shift (i.e., in Canada, there has been growth in immigrants from Asia and a decline of immigrants from Europe) [31]. Immigrants are a heterogenous group who arrive with different life experiences and skills, and encounter varied post-migration barriers to living, working and socializing [32]. It is necessary to consider the differential influence of these factors on the development of loneliness in immigrant sub-groups and to have national surveys that capture these sub-groups in sufficient numbers to enable robust analyses.

We found increased length of time in Canada to be associated with decreased odds of loneliness. Previous studies have found either no association or a positive association between length of time in the host country and loneliness [33–35]. Our finding is supported by qualitative research, in which increased time in the host country means more time to obtain resources (e.g., money, language skills) and build social support required for participation in many activities [36]. This may also explain why we did not find increased risk of loneliness for older immigrants, given that a large proportion of our sample were immigrants with long duration of time in Canada.

Sex and health were two important individual-level correlates, irrespective of immigrant status. Older Canadian-born women had 1.5 times greater odds of loneliness than men, and older immigrant women saw 1.4 times the odds of loneliness. This finding is similar to that of previous studies [37]. Women face greater exposure to key risk factors such as increased caregiver burden and widowhood but lower income compared to men [38–41]. Additionally, women are more likely to be candid regarding their feelings of loneliness than men [42]. Poor self-perceived health was a significant correlate in older immigrants, while poor self-perceived mental health was a significant correlate in older Canadian-born individuals. While previous studies have found that health and mental health is associated with loneliness in older adults generally [14, 20, 43], our finding provides insight into the different impact of health on the two diverse groups.

Relationship-level factors are critical to understand in the process of identifying feasible and immediate ways to address loneliness. In both groups, we observed associations between loneliness and positive social interaction, as well as wanting to participate more in social, recreational and group activities. This finding supports results from previous studies that demonstrate the importance of quality over quantity of social interaction in protecting against loneliness [9, 44]. Living alone was associated with increased loneliness only among Canadian-born older adults, although previous studies generally found that living alone increases risk of loneliness in both immigrants and Canadian-born individuals [33, 44, 45]. However, some immigrants choose to live alone to avoid being a burden to their children [46], and this personal choice may lead to protection against loneliness [47].

Our findings show that, in the development of interventions aimed at fostering social relationships, it is important to acknowledge individual preferences and gaps in social interaction. For example, many older immigrants live in intergenerational homes but studies have shown that this is only protective against loneliness if the relationships are perceived as positive [48]. Non-kin relationships have also been found to be of great value to immigrant populations relative to kin relationships [42]. This suggests the need to consider the heterogeneous nature of older adults and older immigrants in order to implement tailored solutions that will increase opportunities to establish what the *individual* deems as high-quality connections.

Community-level correlates were only significant among older immigrants. Older immigrants living in areas of higher income and ethnic diversity had decreased loneliness. This may be a result of increased access to social activities due to greater resources, on top of living in areas with more linguistically and culturally matched peers and programs [42, 49]. There is currently limited research that explores how the social environment impacts immigrant loneliness. Further research is needed to first, understand how one’s environment creates and perpetuates inequities in immigrant loneliness, and second, support upstream interventions to mitigate such inequities.

Our study strengths include use of a population-based sample, linkage to health administrative data to evaluate the relationship between neighbourhood-level factors and loneliness, and use of a reliable and validated measure of loneliness. Our study also has limitations. The results of our study may not be fully reflective of the current immigrant profiles, loneliness prevalence and risk factors as the data was collected in 2008–2009, which was the most current data source on loneliness linkable to health administrative data in Ontario. Statistics Canada did not report response rates by immigrant status, however, we acknowledge that marginalized immigrants, such as those who do not speak English or French, are more likely to be underrepresented in the CCHS-HA sample [50], despite being more likely to experience loneliness [9]. This could potentially introduce biases into our study if the characteristics associated with non-response differ between immigrant and non-immigrant populations. Further research is needed to explore the extent and nature of non-response bias in surveys of immigrant populations, and to identify strategies to increase representation of underrepresented groups. This analysis also does not consider that respondents within the same neighbourhood may have outcomes that are correlated with one another, which may have overestimated the precision of estimates. Furthermore, due to sample size limitations, we were also unable to stratify immigrants by potential moderators such as immigrant type and ethnic group. Some variables were also not collected, variables such as peri-migration experiences (e.g., discrimination), which could have provided more insight into the risk factors of loneliness. Therefore, it will be important to repeat analyses over time as well as develop and use surveys with more comprehensive data collection to better understand immigrants and their experiences with loneliness.

## Conclusion

This study offers an understanding of the correlates of loneliness among immigrant and Canadian-born adults in Canada at three levels of influence. Future interventions for older immigrants should prioritize the development of individualized services, especially for recent immigrants from countries of greater cultural and linguistic distance. It is critical to continue applying specialized knowledge of loneliness in this population to inform future research and program development that will address systemic issues negatively affecting immigrant communities.

## Electronic supplementary material

Below is the link to the electronic supplementary material.

Supplementary Material 1

## Data Availability

The dataset from this study is held securely in coded form at ICES. While legal data sharing agreements between ICES and data providers (e.g., healthcare organizations and government) prohibit ICES from making the dataset publicly available, access may be granted to those who meet pre-specified criteria for confidential access, available at www.ices.on.ca/DAS (email: das@ices.on.ca). The full dataset creation plan and underlying analytic code are available from the authors upon request, understanding that the computer programs may rely upon coding templates or macros that are unique to ICES and are therefore either inaccessible or may require modification.

## List of abbreviations

CCHS-HA: Canadian Community Health Survey - Healthy Aging
PHIPA: Ontario’s Personal Health Information Protection Act
REB: Research Ethics Board
