# Supplementary material for: Factors associated with loneliness in immigrant and Canadian-born older adults in Ontario, Canada: a population-based study
Source: BMC Geriatr. 2023 Jun 21;23:380. doi: 10.1186/s12877-023-04092-w (PMC10286390; doi:10.1186/s12877-023-04092-w)
Supplement: Supplementary file 1 — Supplementary Material 1 [file 12877_2023_4092_MOESM1_ESM.docx]

**Appendix A: Study Exclusions Flow Chart**

2,680 individuals

5,020 individuals

5,042 individuals

5,140 individuals

98 excluded for:

- Non-Ontario resident as of index date
- Nonsensical death date before index date
- Proxy interview noted

9 excluded for: Missing loneliness data

2671 individuals

- 1703 Canadian-born
- 968 Immigrant

22 excluded for: History of long-term care admission

2,340 excluded for: Age < 65 years as of index date

**Appendix B: Data Sources and Definitions of Descriptive Characteristics**

| **Characteristic** | **Data Source** | **Definition** |
| --- | --- | --- |
| **Individual-Level Factors** | | |
| Age | RPDB | Calculated as of the interview date, based on date of birth in the Registered Persons Database (RPBD). |
| Sex | RPDB | Sex was determined based on the Registered Persons Database (RPBD) as female or male. |
| Immigration status | CCHS | (var SDCFIMM) Respondents were asked on the CCHS whether they were born a Canadian citizen and classified as Canadian-born if they responded ‘Yes’ and as an immigrant if they responded ‘No’. |
| Marital status | CCHS | (var DHH_MS) Respondents were asked about marital status (married; living common-law; widowed; separated; divorced; single, never married), and categorized as: married or common-law; widowed; separated or divorced; single, never married. |
| Education level | CCHS | (var EDUDR04) Respondents were asked to indicate the highest level of education they acquired and categorized as: less than secondary school; secondary school; some post-secondary school; complete post-secondary school. |
| Ethnicity | CCHS | (var SDCDCGT) Respondents were asked to indicate their cultural/racial origin and categorized as: White or visible minority. |
| Self-perceived health | CCHS | (var GEN_01) Respondents were asked to rate their health as: excellent, very good, good, fair or poor. This variable was categorized as: good/very good/excellent, fair/poor. |
| Self-perceived mental health | CCHS | (var GEN_02B) Respondents were asked to rate their mental health as: excellent, very good, good, fair or poor. This variable was categorized as: good/very good/excellent, fair/poor. |
| Number of primary care visits in the past year | OHIP | Counts the number of visits where the specialty indicated is GP/FP (general or family practitioner) or community medicine (and excludes lab services). |
| Have a valid driver’s license | CCHS | (var TRA_01) Respondents were asked if they have a valid driver’s license, and categorized as: yes, no. |
| Country of birth | CCHS | (var SDC_1) Self-reported country of birth, categorized as: US/UK/France, other Europe, East Asia, South Asia, Southeast Asia, South America, Caribbean, other. |
| Age at Immigration | CCHS | (var SDCDAIM) Respondents were asked their age at the time of immigration (in years). |
| Time in Canada | CCHS | (var SDCDRES) This variable indicates the length of time in years the respondent has been in Canada since their immigration and was calculated as the interview date minus the immigration date (in years). |
| Language Able to Conduct a Conversation | CCHS | (var SDCDLNG) This variable indicates the language(s) in which the respondent can converse and was grouped as: English or French only; English or French and other; neither English nor French. |
| **Relationship-Level Factors** | | |
| Positive social interaction | CCHS | (var SSADSOC) This variable is one of four categories of social support measured by the Medical Outcomes Study (MOS) Social Support Survey and measures the availability of other persons to positively interact with. Questions are included about whether the respondent has someone to have a good time with, get together with for relaxation, do things with to get their mind off things or someone to do something enjoyable with. The scale ranges from 0 to 16, with higher scores indicating higher level of positive social interaction. |
| Frequency of community-related activity participation | CCHS | (var SPAGFRE) This variable categorizes respondents by the frequency of their participation in any type of community-related activity during the past 12 months, which is grouped as: did not participate (none), participated at least once a year (yearly), participated at least once a month (monthly), participated at least once a week (weekly), participated at least once a day (daily). |
| Wanted to participate in more social, recreational or group activities | CCHS | (var SPA_Q09) Respondents were asked if they felt like they wanted to participate in more social, recreational or group activities, which was categorized as: yes, no. |
| Sense of belonging to the local community | CCHS | (var GEN_10) Respondents were asked to describe their sense of belonging to their local community, and categorized as: strong (very or somewhat strong), weak (very or somewhat weak). |
| Household size | CCHS | (var DHHDHSZ) Respondents were asked to report a count of the number of people living within the household. |
| Living arrangement | CCHS | (var DHHDLVG) Respondents were asked about the people in their household (if applicable) and categorized as: living alone, living with others. |
| Type of dwelling | CCHS | (var DHHDDWE) This variable indicates the type of dwelling the respondent lives in, and was grouped as: single detached, apartment, mobile home, other multiple dwelling unit, other. |
| **Community-Level Factors** | | |
| Neighbourhood dependency score | RPDB  PCCF  Ontario Census Area Profiles (2006) | A dimension of ON-MARG measuring people who do not have income from employment (e.g., older adults, children). Measured at the dissemination area level, using information from the 2006 Canadian census and weighted using weights obtained to calculate factor scores, which are standardized across Canada. Quintiles were created by placing 20% of the geographic units in the province into each quintile. 1 indicates neighbourhoods of lowest dependency score, while 5 indicates neighbourhoods of highest dependency score. |
| Neighbourhood ethnic diversity | RPDB  PCCF  Ontario Census Area Profiles (2006) | A dimension of ON-MARG measuring concentrations of recent immigrants and/or racialized persons. Measured at the dissemination area level, using information from the 2006 Canadian census and weighted using weights obtained to calculate factor scores, which are standardized across Canada. Quintiles were created by placing 20% of the geographic units in the province into each quintile. 1 indicates neighbourhoods of lowest ethnic diversity, while 5 indicates neighbourhoods of highest ethnic diversity. |
| Neighbourhood income quintile | RPDB  PCCF  Ontario Census Area Profiles (2006) | Estimated by linking an individual’s postal code to existing average household income data for their neighbourhood and dividing into quintiles to generate neighbourhood-specific income quintiles. 1 indicates neighbourhoods of lowest average income levels, while 5 indicates neighbourhoods of highest average income levels. |
| CCHS = Canadian Community Health Survey; OHIP = Ontario Health Insurance Plan Claims Database; ON-MARG = Ontario Marginalization Index; PCCF = Postal Code Conversion File; RPDB = Registered Persons Database. | | |
